# Supplementary material for: Are carbon emissions trading and green financial instruments synergistic? -Comprehensive quantitative research based on content analysis
Source: PLoS One. 2024 Mar 7;19(3):e0298601. doi: 10.1371/journal.pone.0298601 (PMC10919601; doi:10.1371/journal.pone.0298601)
Supplement: S1 Data — (ZIP) [file pone.0298601.s001.zip › Data/地级市Z_F工作报告.zip!/┴╔─■/│»╤⌠╩╨/└┤╫╘╣1⁄2╓┌║┼í╛┬φ┐╦╩2╛▌═°í┐_2006.docx]

各位代表：

我代表市人民政府向大会报告工作，请予审议，并请各位政协委员和其他列席人员提出意见。

“十五”时期的工作回顾

五年来，在省委、省政府和市委的正确领导下，全市各族人民以邓小平理论和“三个代表”重要思想为指导，聚精会神搞建设，一心一意谋发展，胜利完成了市七届人大三次会议通过的“十五”计划各项任务，全面实现了市八届人大二次会议确定的2005年各项预期目标，经济快速发展，事业不断进步，社会保持稳定，改革开放和现代化建设进入了一个新的发展阶段。

——经济综合实力显著增强， 经济格局基本实现了由农业主导型向工业主导型的跨越。 2005年地区生产总值预计实现215.5亿元， 比上年增长17.5%， 五年翻了一番多。 财政一般预算和国地税收入分别实现10.9亿元和28.5亿元， 比上年增长37.5%和31.5%， 按可比口径五年分别增加1.9倍和1.5倍。 经济结构出现可喜变化， 三次产业比由“九五”期末的20.7∶32.7∶46.6调整到24.8∶40.4∶34.8。凌钢新区等一批重点项目启动建设，骨干企业群体不断壮大，工业增加值占生产总值的比重由“九五”期末的23.5%提高到35.6%，以工业为主导的经济格局初步形成。农业结构调整取得明显成效，经济增长方式发生巨大转变，农业三大主导产业产值比上年增长35.7%，五年增加2.3倍；粮食总产达到41.3亿斤，创历史最高水平；农业产业化经营实现较大突破，龙头企业由“九五”期末的45户增加到103户。第三产业持续发展，社会消费品零售总额达到64.1亿元，比上年增长14.1%，五年年均增长11.4%。县域经济增势强劲，生产总值比上年增长23%；北票市、凌源市、朝阳县和建平县财政收入均超亿元，五县（市）财政收入在全省44个县（市）排名中位次整体前移。

——改革开放取得重大突破，社会主义市场经济体制初步建立。国有企业产权制度改革取得重大成果，市直国有工业企业和非工业企业改制面分别达到97.8%和94.2%，县属工业企业基本完成改制任务，改制后的大部分企业焕发生机活力。所有制结构调整快速推进，2005年民营经济营业收入、增加值、税金分别实现265亿元、60亿元和11亿元，比上年增长29.2%、20%和48.6%。社保体系改革取得较大成就，五年共并轨职工16.5万人，养老保险、医疗保险和享受最低生活保障人数分别比“九五”期末增加4.6万人、27.3万人和6.2万人，资金发放率均达100%，失业保险、工伤保险和生育保险稳步推进。农村税费改革顺利进行，农民实际负担水平降至零；粮食流通体制、农村土地流转、“四荒”出让、水利和林业产权制度等项改革深入开展。事业单位改革全面展开，市县两级中介类和经营类单位全部划出事业单位管理序列；乡镇涉农文化类事业单位改革取得实质性进展，机构、人员均减少50%以上。教育改革扎实进行，建立了以县为主的农村义务教育管理体制，教师工资实现统筹、正常发放，中小学人事制度改革基本结束。市县两级政府机构改革全部完成，行政审批制度改革进一步深化，审批项目由“九五”期末的1363个减少到318个；行政许可项目进厅率、办结率分别达到90.4%和99.7%，步入全省先进行列；取消市级收费项目253项,降低收费项目112项，共减轻企业和群众负担2.04亿元。广电资源得到整合，卫生、财政、金融、国有资产管理等改革不断深化。开放型经济实现较快发展，对外开放水平不断提高，五年累计实际利用外资1.05亿美元，是改革开放以来利用外资最多的时期；对内开放力度明显加大，一大批企业落户朝阳，2005年吸引域外资金50亿元，比上年增长26.7%，五年累计吸引域外资金129.5亿元，年均增长25%；出口创汇实现2亿美元，比上年增长51.5%，五年年均增长44.7%。调整理顺了市开发区管理体制和行政区划，开发区入驻项目总投资4.1亿元，比上年增长41%，近三年累计投资11.24亿元，是建区前10年投资总和的13倍。各县（市）区项目区建设从无到有、从小到大，近两年累计完成基础设施投资1.32亿元、引进企业58户，产业聚集和招商引资平台作用日益增强。

——基础设施建设力度空前，城乡面貌发生深刻变化。2005年完成全社会固定资产投资84亿元，比上年增长30%，五年累计完成277亿元，相当于前三个五年计划期的总和。中心城市扩容提质成果显著，面貌大为改观，城市“东进”初见成效，北大街棚户区改造、双塔古街工程已具雏形，大凌河城区段综合整治一期工程全面竣工、二期工程进展顺利，第一高中、凌凤大桥、体育场等重点项目建成并投入使用；城市“西拓”拉开序幕，市开发区以园扩城成效明显，西部工业园区开始规划建设；中心商业区发展步伐加快，燕都国际酒店、火车站候车楼、人民会堂等一批基础设施建筑落成使用，五年共新建和改造道路120条（处），人均拥有道路、绿地由“九五”期末的5.5平方米、5.3平方米分别提高到7.8平方米和6.9平方米。社区建设全面加强，办公用房问题得到基本解决。环境保护力度加大，城区空气质量全年1/2天数基本达到国家二级标准。北票、凌源、建平、喀左四座县城五年新增建成区面积10平方公里,重点小城镇建设稳步推进。锦朝高速公路建成通车，五年累计新增公路里程343公里，实现了全部乡镇和50%的村通油路。山区和农田基本建设扎实进行，我市连续获得“大禹杯”。完成造林90.4万亩，五年累计造林合格面积344.7万亩，森林覆盖率比“九五”期末提高2.5个百分点。城乡电网改造全面完成。固定电话、移动电话分别由“九五”期末的31万户、13万户增加到79.5万户和63万户，国际互联网用户发展到3.98万户。

——人民群众得到较大实惠，生活质量稳步提高。2005年城镇居民人均可支配收入、农民人均纯收入分别达到6600元和3002元，比上年增长16.1%和24.3%，五年年均分别增长10.4%和24.4%。农业税的全部免缴和对农村转移支付补助的增加，惠及了247万农民。城乡居民储蓄存款余额达到218亿元，比上年增长13.1%，五年年均增长14.1%。城市和农村居民人均居住面积分别达到17.74平方米和24平方米，比“九五”期末增加5.07平方米和3.9平方米；北票和市区北大街、锦承铁路两侧棚户区改造工程进展顺利；实施北票采煤沉陷区治理，620户群众搬入了新居。全力推进就业和再就业工作，五年安置18.3万人，其中安置“4050”人员1.4万人，输出劳务130万人次。城市贫困户取暖保障机制基本建立，近三年发放供暖补贴3000万元。加强农村饮水井和防氟改水井建设，10.75万名高氟水重病区群众喝上了安全水。深入开展扶贫开发和扶贫帮困活动，五年累计446个村实现整体脱贫，45万农户拥有稳定收入项目，贫困人口由“九五”期末的120万人减少到30万人，临时救助困难群众50多万人次，资助贫困学生35万人次；2005年全市发放农村义务教育“两免一补”资金2977万元，9万名困难学生受益，为5200户无电视农户捐赠了电视机。近两年共筹措资金2.4亿元，提前全部还清了拖欠公教人员标准工资。市政府在八届人大二次会议上承诺的九件实事基本落实。

——科教兴市战略深入实施，各项社会事业取得新的成就。2005年高新技术产品实现产值32亿元，比上年增长25%，年均增长20%；五年共有132项科研成果达到国内领先或先进水平，42项获省政府科技进步奖。五年共建农村九年一贯制学校31所，改造危房44.1万平方米；新建普通高中6所，在校生由“九五”期末的3万人扩大到6.4万人；新建和改建城区小学6所，城区小学大班额问题得到基本解决；中等职业教育改扩建校舍21.2万平方米，朝阳大学开始规划，教育事业整体步入全省前列。制定人才培养、引进、使用和管理的优惠政策，人才队伍不断壮大。话剧《凌河影人》获得国家舞台艺术精品工程十大精品剧目殊荣，以“文化下乡”、“凌河之夏艺术节”、“少儿艺术节”为代表的社会文化活动日趋活跃。中心医院、二院门诊楼建成并投入使用，五个县（市）医院门诊楼和病房楼全部进行了改扩建；疾病防控工作得到加强，完成了市县两级疾控中心建设改造，建筑面积全部达到或超过国家标准；北票新型农村合作医疗试点任务全面完成。全民健身活动更加普及，我市连续被评为全省群众体育工作先进市和优秀市；竞技体育运动水平不断提高，五年共获得国家级以上比赛金牌57枚。宗教政策得到较好落实，民族地区发展水平迅速提升，我市民族工作获得国务院表彰。计划生育、土地管理工作取得显著成绩，各项人口控制指标全部完成，耕地总量实现了动态平衡。如期完成全国第一次经济普查规定的各项任务。新闻出版、档案、残疾人保障、人防、气象、防震减灾抢险、地方志、老龄、妇女儿童等项事业均有长足进步。

——精神文明建设取得丰硕成果，民主法制建设得到新的加强。以打造“信心朝阳”为目标，通过广泛开展学赤峰、促振兴活动和学习冀鲁先进经验，推动了新一轮思想解放。认真贯彻《公民道德建设实施纲要》，大力加强了社会公德、职业道德、家庭美德教育和未成年人思想道德建设，公民思想道德素质明显提高。深入开展了群众性精神文明创建活动，五年间获得国家和省表彰的文明单位分别达到15个（次）和324个（次）。强化全民国防教育，狠抓民兵和预备役工作，提高了军警民共建水平。坚持并完善向人大报告工作、向政协通报情况制度，自觉接受人大及其常委会的法律监督和工作监督，重视发挥政协的政治协商、民主监督和参政议政作用，提高了政府决策科学化、民主化水平；五年共办理人大代表建议342件、政协提案797件，办复率均达100%，满意率均在95%以上。政府法制建设水平不断提高，行政执法、行政复议等工作走上规范化轨道。推行政务、厂务和村务公开，民主政治建设得到加强。积极做好信访和人民调解工作，认真落实领导包案下访等措施，着力解决信访突出问题，妥善处理了人民内部矛盾。坚持依法治市，“四五”普法顺利开展。强化社会治安综合治理，依法严厉打击了各类犯罪活动。整顿规范市场经济秩序和矿业秩序，安全生产形势逐步好转。

各位代表！回顾“十五”，全市人民走过了一条不平凡的道路，经历了体制转轨期间矛盾凸显和宏观环境趋紧的艰苦磨练，战胜了连续三年严重干旱等自然灾害，经受住了防控非典和禽流感的严峻考验。五年来，我们不断深化对市情的认识，准确把握朝阳所面临的形势，坚定不移地把发展作为第一要务，努力抢抓机遇，靠新观念加快发展，靠新思路指导发展，靠硬措施推进发展，使我市经济社会全面进步；坚定不移地把改革开放作为第一动力，努力用体制机制创新激发了经济活力，用对内对外开放拓展了发展空间；坚定不移地实施项目支撑战略，形成了全局工作以经济建设为中心、经济建设以项目建设为中心的工作机制，用项目的生成和壮大增强了经济发展后劲；坚定不移地以人的全面发展为根本，既紧紧依靠人民群众，又时刻关注民生、了解民意、集中民智、珍惜民力，形成了加快发展的强大合力；坚定不移地转变作风、真抓实干，进一步提高工作效率，优化经济发展软环境，努力塑造对人民高度负责的政府新形象，推进了全市物质文明、政治文明、精神文明的协调发展。五年来的实践，既为朝阳今后发展奠定了坚实的物质基础，也提供了良好的工作基础和思想基础。这些成绩的取得，是市委正确领导的结果，是市人大及其常委会和市政协监督支持的结果，是全市上下励精图治、顽强拼搏，社会各界齐心协力、鼎力相助的结果。在此，我代表市人民政府，向全市广大工人、农民、知识分子和干部，向驻朝解放军指战员和武警官兵，向各民主党派、工商联、各人民团体和各界人士，向中央和省驻朝单位，向所有关心和支持朝阳发展的朋友们，表示衷心的感谢和崇高的敬意！

在肯定成绩的同时，我们也清醒地看到，我市经济社会发展还存在许多困难和问题。主要是：经济总量小，投入能力差，群众生活还比较困难；经济结构不够合理，工业的主导地位还不稳固，三次产业内部存在较大结构缺欠，产业整体规模不大、竞争力不强；经济社会发展还面临诸多体制机制性障碍，改革成果仍处于较低层次，部分领域改革滞后；资源利用率低，存在着大量闲置与严重浪费并存的问题，经济发展与资源环境的矛盾日益突出；高新产业发展迟缓，产业升级的科技动力不足，自主创新工作基础薄弱；城镇化水平落后，支撑带动和集聚辐射能力不强，制约了经济社会的发展；对外开放水平不高，经济外向度明显低于全省平均水平。这些困难和问题，有的是长期积累并需长期努力才能解决的，有的是体制转轨和结构调整过程中难以避免的，也有的是政府工作中的缺点和不足造成的。尽管我们在政府自身建设方面做了不懈努力，但政府职能转变还未完全到位，“越位”、“缺位”和“错位”情况时有发生，在政府部门以及工作人员中，服务意识不强、依法行政水平不高、作风不实的问题还没有得到很好解决，特别是发展软环境还不优，存在办事拖拉、吃拿卡要等现象。我们一定高度重视这些问题，采取综合措施，切实加以解决。

“十一五”发展目标及2006年主要工作任务

各位代表，国家深入实施振兴东北老工业基地战略，强力推进环渤海湾地区开发开放，全省进一步落实开发辽西北各项政策，这都充分表明，今后五年将是朝阳本世纪头二十年重要战略机遇期中尤为关键的时期。尽管前进的道路上充满着激烈的竞争和严峻的挑战，但是，只要我们审时度势，积极应对，艰苦奋斗，勇于开拓，就一定能够变挑战的压力为前进的动力，变机遇的潜力为发展的实力，完全可以实现更快、更好的发展，完全可以使“十一五”时期，成为朝阳经济社会发展的跨越期、综合实力横向比较的超越期和城乡形象的提升期。

为了把今后五年朝阳经济和社会发展的蓝图规划好、设计好，我们制定了《朝阳市国民经济和社会发展第十一个五年规划纲要（草案）》，提请本次大会审议。今后五年，我们要完成基本实现朝阳老工业基地振兴，实现整体稳定脱贫并向全面小康社会迈进两大战略任务，到2010年，地区生产总值年均增长17%左右，人均生产总值比2005年翻一番；财政收入年均增长18%；固定资产投资五年累计完成1000亿元，年均增长30%以上；城镇居民可支配收入、农民人均纯收入年均分别增长12%以上；万元生产总值综合能耗比“十五”期末降低20%；高新技术产业增加值占全市工业增加值比重达到20%以上；社会事业整体水平实现快速提高，基本达到或接近全省平均发展水平。经过五年的奋斗，全市经济社会发展将呈现新的局面：一是经济结构的战略性调整取得显著进展，基本实现老工业基地振兴，形成更具活力、更加开放的经济体系，综合竞争实力显著增强；二是市场取向的改革向纵深推进，形成更加完善的社会主义市场经济体制；三是城镇体系更加健全，城市功能不断完善，社会主义新农村建设取得明显进展，形成富有人居引力和创业引力的良好环境；四是人民生活质量明显提高，农村贫困人口完成由贫困向温饱的历史性跨越，社会全面进步，为全面建设小康社会奠定基础。《纲要（草案）》一经大会审议通过，我们将认真组织实施。

2006年，是“十一五”的开局之年。做好今年工作，关乎全局，意义重大。各级政府要认真贯彻落实市委七届十一次全会精神，坚持以科学发展观统领经济社会发展全局，坚定不移地实施“一主四化”战略，努力实现经济社会又快又好的发展，实现“十一五”的高水平开局。主要预期目标为：地区生产总值增长17%，地方财政一般预算收入增长18.5%，全社会固定资产投资增长40%，城镇居民人均可支配收入和农民人均纯收入分别增长12%，城镇登记失业率控制在6%以内，各项社会事业与经济建设协调发展、同步提高。

为了确保实现上述目标，我们要全力做好以下六个方面重点工作：

 一、推进结构调整和增长方式转变，壮大经济综合实力

提高产业竞争力。推动工业结构优化升级，保持工业经济快速增长势头，确保工业增加值增长21%。以培育优势产业为目标，以提高资源配置和利用效率为重点，把优势产业的发展方向集中到打造冶金业、汽车工业、农产品加工业、矿产资源加工业、纺织业和旅游业资源深度开发链条上，加快朝阳由资源大市向产业大市转变。大力发展装备制造业，认真研究制定相关配套措施，争取在重型汽车组装和冶金成套设备制造领域率先实现突破，提高装备制造业在工业经济中的比重。制定实施全市循环经济发展规划，开展节能、节水、节地、节材工作，力争在钢铁、建材、能源等重点工业领域实现循环经济发展的实质性突破。积极开拓域内外建筑市场，加快发展建筑业。大力发展现代农业，突出畜牧业在农村经济中的第一主导产业地位，切实做好高致病性禽流感等重大动物疫病防控工作，加快畜禽品种改良步伐，新发展一批畜牧专业小区、专业村屯和大场大户，扩充商品量，提高附加值，畜牧业产值占农业总产值比重达到48%左右；继续优化保护地种植结构，搞好新品种引进和试验示范工作，新增保护地6万亩以上；积极发展林果业和林下经济，提高产出水平和经济效益；大力发展蔬菜、花卉、水果、棉花、粮饲兼用玉米、杂粮等特色农业；着力搞好无公害、绿色、有机农产品的标准化生产；培育发展农民合作经济组织，切实提高农民的组织化程度；加大对农产品深加工企业的支持力度，争取更多的企业进入省级农业产业化龙头企业行列，农产品加工产值增长20%以上。加快现代服务业发展步伐，搞好第三产业发展规划，制定出台强有力的推进措施。认真实施《朝阳市旅游发展总体规划》，进一步整合和深度开发旅游资源，加大旅游市场开发力度，合理配置旅游要素，争创中国优秀旅游城市；推进金融业改革和发展，防范金融风险，引导金融机构加大对地方经济发展的支持力度，组建完成城市商业银行，发展壮大农村信用社；大力发展流通业，探索建立农村新型流通体制，培育壮大一批具有较强集散能力的特色市场，提升中心城市商贸核心区的整体形象；加快发展房地产业，积极拓展现代信息、中介服务、邮政通信等产业领域，大力发展连锁经营、物流配送、电子商务、特许经营、社区超市等现代流通业态。社会消费品零售总额增长14%。

提高项目支撑力。进一步强化全局工作以经济建设为中心、经济建设以项目建设为中心的工作机制，再掀项目建设新高潮，确保开工建设投资1000万元以上项目260个左右，完成项目储备400个以上。举全市之力，加快推进凌钢新区建设，基本完成各项规划设计、主体工程土建和设备采购任务；集中力量抓好京四高速公路朝阳段、朝赤高速公路、“燕水北调”、朝柴10万台轻型车用柴油机、浪马100万套扩产改造、中泽朝纺10万锭易地扩建、飞马重型汽车制动盘、凌汽重型汽车组装生产线、兰凌日产5000吨水泥等重点项目建设，力争燕山湖发电厂项目获得核准。

提高企业成长力。以市场为导向，以产品为龙头，以增强自主创新能力为方向，集中力量扶优培强一批重点骨干企业，初步形成立市立县企业群体，新增超5000万元企业10户、超亿元企业8户。积极引导和支持中小企业，向“专、特、优、新”方向发展，增强为骨干企业配套服务能力，新增各类规模以上企业100户。调整科技资金投向，大力创办和引进一批高科技企业。加快建设以企业为主体的技术创新体系，着力扶持企业科研所的发展 ，选择5家基本具备条件的企业，开展自主创新试点工作。引导企业走品牌兴业之路，下大气力培育发展名牌产品。

提高财政保障力。把增加财政税收作为壮大经济整体实力的重要任务来抓，坚持科学生财，继续巩固现有财源，培育新兴财源，开发后继财源，综合运用税收、财政贴息等手段，加大对重点企业和纳税大户扶持力度，提高企业创税能力。坚持科学聚财，进一步完善税收和非税收入的征缴办法和手段，努力实现应收尽收；继续加强国有资产经营工作，确保国有股权收益和经营性收入全部纳入财政；积极争取上级财政转移支付和各类专项补助资金，切实增加政府可支配财力。坚持科学理财，按照公共财政框架的要求规范供给范围，调整优化支出结构，保障工资、社保等重点需求，发挥审计和财政监督职能，把好财政支出关；全面推进国库集中收付制度改革、政府采购制度改革，加强绩效考核，提高财政资金的使用效益。

二、以发展县域经济和推进城镇化为重点，加快建设社会主义新农村

大力发展县域经济。把建设社会主义新农村的着力点放在大力发展县域经济上，坚持走以工促农、以城带乡，以工业化为主导，以农业现代化为基础的新农村建设之路。强力推进县域经济特色化，特色经济产业化，产业发展上规模、上水平，做大做强特色板块经济，确保县域经济发展速度达到20%以上，财政一般预算收入增长25%左右。全面落实鼓励县域经济发展的各项政策措施，进一步放权搞活县域经济；积极开展超亿元产值、超千万元财政一般预算收入、超5000元农民人均纯收入的“三超”乡镇达标和“十强”乡镇评比活动，激发乡镇经济的发展活力。高质量做好农业普查工作，为发展农村经济提供科学依据。

加快推进城镇化步伐。拓展城镇发展空间，进一步扩大建成区规模，全市城镇化率确保提升2个百分点以上。制定和完善包括凤凰组团修建性详规、柳城组团总规和燕都组团控规在内的各项规划，全面启动县城和县域城镇体系的规划及修编，同时做好土地利用规划调整工作，启动重点小城镇和新农村试点村的规划工作。中心城市按照“沿河发展、组团开发、扩容提质、完善功能”的思路，启动建设凤凰组团基础设施，高标准完成凌凤大街改造；按照“扩容路先行”的要求，启动“内环”公路建设；推进供排水、供电配套设施建设，为凌钢新区建设提供有力支持保障。建设体育馆、文化大厦和古生物化石地质公园标志性建筑，完成大凌河城区段二期改造、双塔古街、天心湖、北大桥等重点工程，启动建设市污水处理厂，建设医疗垃圾无害化处理站和安全转运站。建立和完善城市管理长效机制，加大综合执法力度，全面消灭中心城区所有地上垃圾箱，争取城市管理工作进入全省先进行列。进一步加强城市土地出让工作，增加土地收益。盘活城市各类资产，鼓励引导社会力量参与城镇基础设施、公用设施建设。把县城建设摆在更加重要的位置，坚持新区开发与旧城改造并举，加大基础设施投入力度，提升整体形象；朝阳县要在规划新区内全面启动基础设施建设，打造高标准项目区，为县城搬迁创造条件。把重点小城镇建设作为今后一个时期的工作重点，明确发展定位，加快改造步伐，全方位改善面貌。以两条高速公路建设为契机，提前做好县城及相关城镇出口路的规划建设工作，努力实现以路扩城。落实户籍、就业、社保等相关配套政策，逐步消除阻碍城镇化发展的体制性障碍。

实现园区建设新突破。认真编制好中心城市西部工业园区的总体规划及其各专业园区规划，进一步细化各县（市）区项目区详规，搞好辐射辽冀蒙三边地带的物流园区规划。牢固树立“举债兴业、设施先行”的思想，通过多渠道融资、多元化投入，搞好园区水电路等基础设施建设，尽快完善硬件环境。运用相关政策加大土地储备力度，着力解决好开发区和项目区的建设用地问题。进一步完善园区管理体制和运行机制，建立健全利益分配和激励机制，赋予园区更大的经营管理自主权。强力推进产业集群，加大项目向园区集聚力度，实现以园兴工、以园扩城目标。市开发区累计开发面积达到7平方公里，招商引资和固定资产投入均完成5亿元以上；各个县（市）区项目区建设要实现规划水准、基础设施建设、项目引进和产业聚集的新突破。

改善农村基础设施条件。搞好朝阳客运中心、建平客运站和一批乡间客运站建设，配合铁路部门抓好朝阳段既有铁路提速扩能改造；积极探索建立以“村民自愿、村民自治”为核心的村屯路建设模式，完成47个村、160公里农村公路黑色路面，路基储备1000公里，改善农村交通条件。启动村村通自来水工程，建设各县（市）食品安全检验中心，让全市人民吃上“安全水”和“放心食品”。大力推进广播电视进村入户，扩大有线电视网光缆铺设面。加强农村公共文化建设，启动“乡有文化站、村有活动室、组有文化活动中心户”工程，丰富农民精神文化生活。加快改善农村生产条件，不断提高农业机械化水平，坚持不懈地推进山区建设和农田基本建设，搞好为高效农业配水配电、河道治理和“四荒”资源开发，完成水土保持综合治理面积40万亩，新发展农村能源“四位一体”生态农业模式示范户1万户。大力发展农村教育事业，完成50所农村中小学的布局调整工作。以文明生态村建设为载体，每个乡镇至少启动一个试点村，按照先洁后美、先清后改的要求，开展环境综合整治活动，实现村容整洁的目标。

三、再创改革开放新优势，再增加快发展新动力

深化改革，激发经济发展活力。搞好凌钢集团股份制改造、股份有限公司股权分置改革，进一步加大非工口企业改制推进力度，做好国企改制收尾和遗留问题处理工作，全面完成国企改制任务。加快推进交通、城建、水务等市政公用事业改革进程。鼓励和支持民间资本投入社会事业发展，努力实现科技、教育、文化、卫生等社会事业投资主体多元化。积极稳妥地推进行政管理类和公益类事业单位改革，在机构、用人、分配和职称改革方面实现新突破。进一步巩固和扩大农村税费改革成果，全面推进农村卫生改革、市县动物卫生管理体制改革和其他改革工作。继续深化国土资源管理体制改革，落实严格的耕地保护制度，积极推进农村集体建设用地使用权依法流转，鼓励用地者以出让、租赁、作价入股等方式获得土地使用权。

外向拓展，提高对外开放水平。把招商引资作为扩大开放的主攻方向来抓，坚持境外招商与域外招商并重，更多地聚焦域外招商，充分发挥政府的主导作用、各类经济园区的载体平台作用和企业主体作用，围绕现有存量资产、优势资源、企业接长链条、城市基础设施建设、社会事业产业化，有针对性地包装一批重点项目，由各级主要领导带头，以企业招商和专业招商为主力、全民招商团队为补充，开创招商引资新局面，实际利用外资、域外引资要确保分别增长25%和30%以上。继续保持外贸出口的良好态势，在抓好出口大户和大宗产品出口的同时，积极引导各类有条件的企业开拓国际市场，全年出口创汇增长25%以上。

内向激活，加快民营经济发展。认真落实全民创业各项政策措施和领导包扶责任制，加大对全民创业政策和创业典型的宣传，壮大创业主体总量和体量，民营企业和个体工商业户数力争增长20%左右。进一步优化民营经济发展环境，积极发展商会和行业协会，搭建银企沟通平台，认真落实各项扶持政策，鼓励和引导企业不断增投入、上项目、扩规模。在继续壮大市级贷款担保机构实力的同时，完成县级担保机构建设任务，解决好融资难问题。积极支持优势民营企业以控股、收购、联合等方式，参与国有和集体企业的改制改组。民营经济营业收入、入库税金均增长25%以上。

四、关注民生，保障民权，提高人民群众生活水平

千方百计抓好就业。建立健全市场导向、托底安置、城乡统筹、培训联动的就业机制，积极做好普惠制培训工作，全年新增实名制就业4.5万人。大力发展劳务经济，进一步规范覆盖城乡的市、县、乡、村四级劳务输出组织服务网络，实现劳动力市场信息网络乡乡通；进一步强化对输出劳务的技能培训，重点利用现有的职教资源，面向市场和用工单位搞好订单培训、因需施训，完成实名制劳务输出30万人次、有组织劳务输出15万人次以上。

健全完善社保体系。完成基本养老保险市级统筹工作，确保养老金按时足额发放。加大各类保险费的征缴和筹集力度，依法抓好用人单位社会保险参保、缴费和清欠工作，不断扩大社会保险覆盖面。继续抓好低保的分类救助、分类管理工作，实施乡镇企业职工养老保险试点，扩大单纯务农农民的养老保险覆盖面，认真落实被征地农民的基本生活保障。全面实行住房公积金制度，切实保障职工的合法权益。完善城市贫困户取暖长效保障机制，健全城乡困难群众社会救助体系，做好优抚安置、社会救济、社会福利、社会互助和法律援助等工作。

办好涉及人民群众切身利益的10件实事。1、全面完成城市棚户区改造和北票采煤沉陷区治理任务。2、完成200个项目村扶贫和5万个户有稳定收入项目建设，实施移民搬迁1000户。3、全面推行新型农村合作医疗。4、确保有就业愿望的零就业家庭至少一人实现就业。5、对义务教育阶段的所有困难家庭学生实行“两免一补”。6、帮助5000户农村无电视户解决电视机，实现20户以上的自然村屯全部通广播电视。7、全面实施城乡大病救助制度，对市县两级城市低保人口实施医疗救助保障。8、启动八里堡村二期、八宝村、西大营子村、七道泉子南村、榆树林村和肖家村“城中村”改造，争取完成10万平方米以上改造任务。9、进一步改善农村办学条件，新建九年一贯制寄宿学校10所，完成农村中小学危房改造5万平方米。10、严格执行企业最低工资制度，适当提高最低生活保障标准和公务员工资水平。

五、大力发展社会事业，加强精神文明和民主法制建设

努力构建社会主义和谐社会加强精神文明建设，推进社会事业全面发展。深入开展打造“信心朝阳”活动，大力弘扬创业文化，进一步激发广大干部群众的创业精神和信心指数。认真贯彻《公民道德建设实施纲要》，广泛深入开展各种群众性精神文明创建活动。充分发挥省、市科研院所作用，逐步建立符合市场经济要求的新型科研机制；加快市高新技术园区建设，争取进入省级高新技术园区行列。大力发展学前三年教育，巩固和提高普九成果，普及高中阶段教育，加快朝阳本科大学建设步伐，完成资源整合、后勤社会化改革，并实质性启动与其他院校合作建设重点本科工作；加快职教产业化进程，积极构建符合朝阳实际的职业教育发展模式，支持各级各类职业教育办出特点、办出水平，打造职业教育品牌。抓住“培、引、留、用”四个环节，建立完善人才开发的使用机制，加强以优秀公务员、企业经营管理人才和专业技术人才为主体的人才队伍建设。改善城乡医疗卫生条件，加强公共卫生服务体系建设，提升医疗服务质量和突发性公共卫生事件应急能力，完成市传染病医院迁址建设工作；加强对医疗机构的监管，提高医疗质量，降低医疗费用，切实解决好城乡群众“看病难、看病贵”问题。繁荣文艺创作，发展文化产业，壮大文化事业；加强文物保护和开发利用，规范和净化文化市场。大力普及全民健身活动，不断提高竞技体育水平，完成市全民健身活动中心、体校教学楼和综合训练馆建设，在省十运会上争取取得好成绩。认真落实民族宗教政策，依法管理宗教事务，大力支持民族地区经济和社会事业发展。加强国防教育，推进双拥工作。关心支持妇女、儿童、老龄、残疾人事业，进一步做好新闻出版、防震减灾、民兵预备役、气象、档案、地方志等项工作。

实现人口、资源、环境的协调发展。健全计划生育家庭奖励扶助制度，继续开展出生人口性别比失调的专项治理，严格执行人口失控责任追究，提高人口素质，人口出生率控制在10‰以内，计划生育率稳定在98%以上。认真搞好矿业秩序集中整顿，进一步完善矿业权市场建设，积极开展矿产资源地质勘测工作，促进矿产资源深度开发和综合永续利用。进一步保护和开发利用好古生物化石资源。科学利用地下水，大力开发地表水和云中水资源，实现水资源合理开发利用。加快推进生态朝阳建设，完成造林绿化100万亩。积极筹划建设朝阳热电厂，继续实施“拆小并大”工程，拆并小锅炉房20座。加强环境保护与污染治理，确保市区空气质量二级天数全年达到80%以上。

加强民主法制建设。自觉接受市人大及其常委会的法律监督和工作监督、人民政协的民主监督和人民群众的社会监督，提高人大代表的建议、批评、意见和政协提案的办理质量。加强与各民主党派、工商联和人民团体的联系，充分发挥工会、共青团、妇联、科协等群团组织的桥梁和纽带作用。深入开展“五五”普法，突出重点，注重实效，努力增强公民法制观念和依法维权、依法办事的自觉性。深化政务、村务和厂务公开，切实加强基层群众性自治组织建设，大力开展创建和谐社区、和谐村镇活动，全面提高基层民主决策、民主管理、民主监督水平。高度重视群众来信来访，进一步完善和落实信访工作责任制，及时解决群众的合理诉求，积极预防和妥善处置各种信访突出问题和群体性事件。加大社会治安整治力度，积极推进平安市、平安县创建工作，严厉打击各种黑恶势力，依法查处各类治安案件，为经济发展提供安全的社会保障。巩固和完善社会矛盾纠纷排查调解机制，切实提高调解成效。严格落实安全生产责任制，强化对煤矿、非煤矿山、食品、药品、饮食卫生、危险化学品和交通安全等的监管，防止重特大安全事故的发生。坚决打击制假售假、不正当竞争等违法行为，规范市场经济秩序。

六、加强自身建设，打造认真履职、对人民高度负责的政府

转变职能，提高效率。强化政府的社会管理和公共服务职能，改进宏观调控和市场监管方式，把更多的精力放在推进经济社会发展和解决民生问题上。继续深化行政审批制度改革，加快政务信息平台建设，建立健全各种灾害和突发事件应急机制。结合绩效考评，加大对机关效能的奖惩力度，严肃查处“不作为”、“乱作为”现象，消除“中梗阻”，确保政令畅通。大力压缩会议、文件和评比、表彰活动，减少礼仪庆典，简化迎来送往，集中精力抓大事、解难事。

依法行政，科学决策。认真贯彻实施《行政许可法》和《全面推进依法行政实施纲要》，严格依照法律规定的权限和程序行使权力，做到有权必有责，用权受监督，侵权要赔偿，违法要追究。实行行政执法责任制，坚决克服多头执法、执法不公的现象。进一步完善重大决策规则和程序，建立健全公众参与、专家论证和政府决策相结合的民主决策机制，完善人民建议征集制度、社会公示制度和听证制度，提高决策的透明度和公众参与度。

强化责任，狠抓落实。建立科学规范的行政问责制，抓好责任分解、责任考核和责任追究，对每项决策、每个部署都要及时落实反馈和评估，确保各项目标任务落到实处。各级政府组成人员和广大公务员要以夜不能寐、食不甘味的精神和不达目标不罢休的韧劲，切实抓好工作落实。 清正廉洁，优化环境。认真落实党风廉政建设责任制，各级政府及政府部门的主要领导要切实当好“第一责任人”，抓好班子，带好队伍。建立教育、制度、监督并重的惩治和预防腐败体系，对招投标市场、建筑工程、土地出让和国有资产管理等热点领域实施重点监控。进一步加大查处大案要案、纠正部门和行业不正之风工作力度。继续搞好软环境建设，积极开展软环境测评工作，加大涉软案件查处力度，做到有报必查、有查必果、查必到人、查实必处，全力创造支持改革、鼓励干事、保护创业、注重服务的软环境。

建设高素质的公务员队伍。认真学习贯彻《公务员法》，继续推进“五型”机关创建活动，严格实行公务员逢进必考，广泛开展学习培训，不断提高公务员的创新能力和业务素质。进一步加强作风建设，强化认真履职，教育全体公务员始终保持昂扬向上的精神状态，始终保持奋发有为的工作激情，把一切工作的目标指向都聚焦于发展、聚焦于实干，努力塑造为民、务实、清廉的良好形象。

各位代表!我们已经站在新的历史起点上，朝阳正进入一个新的重要发展时期。时代的重任召唤我们奋力前行，人民的期望不容我们丝毫懈怠。让我们高举邓小平理论和“三个代表”重要思想伟大旗帜，以科学发展观为统领，在市委的坚强领导下，紧紧团结和依靠全市各族人民，群策群力，扎实工作，推动经济社会实现跨越式发展，把我们共同热爱的朝阳建设得更加美好！
